# Empathy, psychopathology and suicidal behavior: a case–control study

**DOI:** 10.1186/s12888-025-07230-3

**Published:** 2025-08-26

**Authors:** Elena Toffol, Marialaura Lussignoli, Emanuele Aliverti, Giovanna Capizzi, Igor Bertocci, Paolo Scocco

**Affiliations:** 1 SOPROXI Onlus, Padua, Italy; 2 Department of Public Health, University of Helsinki, Clinicum, PO BOX 20, Helsinki, Finland; 3 Padova Neuroscience Center, University of Padova, Padua, Italy; 4 Department of Statistical Sciences, University of Padova, Padua, Italy; 5 Psychiatric Care Facility Parco dei Tigli, Teolo, Italy

**Keywords:** Empathic, Personal distress, Psychological distress, Attempted suicide, Psychiatric patients

## Abstract

**Background:**

Patients with psychiatric disorders have high levels of self-oriented empathy, but dampened other-oriented empathy. Empathy characteristics in individuals who attempt suicide, and their relationships with psychopathology are not clear.

**Methods:**

Altogether 62 suicide attempters, 64 non-suicidal psychiatric inpatients and 138 healthy controls filled-in self-reported questionnaires on empathy and psychopathology. The relationships between each empathy subscale, levels of psychopathology, and case–control groups were tested via linear regression models and in group-stratified analyses.

**Results:**

Cases had significantly higher Fantasy (FS) and Personal Distress (PD) scores than healthy controls. Higher levels of psychological distress were associated with higher scores of FS (2.10, 1.08‒3.13) and PD (2.90, 1.87‒3.93), irrespective of the group. With increasing psychopathology levels, scores of Perspective Taking decreased significantly in suicide attempters (-1.81, -3.55‒ -0.08), non-significantly in non-suicidal psychiatric inpatients (-1.11, -2.94‒0.73) and increased in healthy controls (0.79, -1.05‒2.64); conversely, PD increased significantly in healthy controls (4.91, 2.86‒6.96) and in psychiatric controls (2.89, 0.95‒4.82), but non-significantly among cases (1.60, -0.13‒3.33).

**Conclusions:**

Empathy does not differ between suicidal and non-suicidal psychiatric patients. Psychopathology is related to empathic PD and FS. The relation is stronger in individuals with no psychiatric conditions than in psychiatric patients or suicide attempters. Emotional and self-oriented dimensions of empathy could contribute to the identification of people at risk of suicidal behavior.

**Supplementary Information:**

The online version contains supplementary material available at 10.1186/s12888-025-07230-3.

## Background

Empathy refers to the individual ability to establish relationships with others, to experience social feelings and to accept the others’ emotionality, while maintaining own boundaries [1]. Thus, empathy can be considered an interpersonal phenomenon, where individuals consciously share and express common feelings without passing judgment [2].

Empathy encompasses two components: while the cognitive component indicates the ability to form a working model of others' emotional states, the affective one refers to the capacity of being sensitive to and vicariously feeling others’ emotions [3]. The individual empathic response may be self- or other-oriented. An empathic response oriented to others prompts an altruistic inclination to help others [4] while taking distance from the situation, and corresponds to the capacity of experiencing and understanding the feelings of others. The Perspective Taking (PT) and Empathic Concern (EC) subscales of the Interpersonal Reactivity Index (IRI), a tool developed by Davis (1983) [5] to assess empathy, clearly cover this dimension. On the other hand, the Personal Distress (PD) and Fantasy (FS) subscales of the IRI embrace the more self-oriented response, where the individual may often lack a buffering distance. Hence, individuals overwhelmed by the exposure to others’ suffering may experience an egoistic motivation to relieve own discomfort, and become themselves at risk for psychological distress [6]. For example, higher levels of self-oriented empathic dimensions (namely, Personal Distress and, to a lesser extent, Fantasy) have been found related to greater posttraumatic stress response and general psychological distress among individuals employed in disaster relief activities, suggesting that those with high self-oriented empathy tend to experience high levels of psychological distress when exposed to others’ suffering [7].

Individuals with depressive symptoms or disorders, as well as schizophrenia and bipolar disorders, display higher levels of self-oriented emotional distress, but possibly lower PT scores [8–12]. In general, affective (specifically, personal distress) rather than cognitive (perspective taking and fantasy) empathy is more altered in depressed patients, with no effect of duration or chronicity of illness [13]. However, in both bipolar and depressive patients, there seems to be a tendency for enhanced PD response in relation to greater severity of depressive symptoms [10, 14].

Empathic dimensions in relation to suicidality and suicidal behavior are less clear. According to interpersonal theories, the development of cooperative behavior and positive relationships relies, among others, on empathic skills. Thus, a suicide attempt may be an alternative way to ask for help in the context of interpersonal difficulties. In particular, feelings of perceived burden towards others and of humbled belonging, along with separated capability and desire to engage in suicidal behavior, may result in a suicidal act [15]. Diminished empathic skills, such as lower empathic perception and the inability to integrate others’ emotions, may contribute to reduce the social deterrent of considering the impact of suicide on family and friends [16], and a failure to recognize others’ emotions may interfere with the capacity to interact with the social environment, thus contributing to a suicidal crisis [17]. In fact, a study of old adults with current or past suicide attempt found lower empathic perception, or a dampened behavioral empathy, in comparison with both depressed non-suicidal patients and healthy controls [16]. On the contrary, schizophrenia patients with a history of attempted suicide were found with higher Fantasy, Personal Distress and, less consistently, Empathic Concern scores than non-suicidal ones [18, 19], with high PD scores remaining associated with suicidal behavior after controlling for covariates, including severity of psychotic symptoms [18]. Similarly, depressed outpatients with a history of non-suicidal self-injury had higher personal distress scores compared to depressed outpatients without such a history, irrespectively of sociodemographic and psychopathologic characteristics [20]. Similarly, we have shown that inpatients who had recently attempted suicide had higher scores at the Personal Distress and Fantasy subscales of the IRI, with no differences in the more other-oriented empathic components, compared to healthy non-suicidal individuals [21]. Considering that the suicidal belief itself (i.e., that others would benefit more from one’s suicide than if he/she continued living), requires consideration of others [15, 22], it is not surprising that individuals who attempt suicide have in fact higher levels of empathy.

Although psychiatric disorders are one of the major risk factors for suicidal behavior, not all psychiatric patients do act suicidal behavior. If empathy dimensions contribute to distinguish suicidal from non-suicidal individuals, irrespective of psychopathology, it has not been examined yet, and our previous study did not consider the role of dimensions and severity of psychopathology in the relation between suicidality and empathy. Thus, this work aims to fill this gap in the existing literature, by specifically addressing the question of whether psychiatric patients who attempt suicide have distinct empathic profiles compared to psychiatric patients who do not, as well as compared to healthy controls. Another main focus is to understand if the relationship between empathy dimensions, and dimensions and severity of psychological distress varies between these three populations. More specifically, this study will test the associations between empathy profiles and suicidal behavior after taking into consideration severity and dimensions of psychopathology, and examine if the relationship between empathy and psychological distress differs between psychiatric patients who attempt suicide, psychiatric patients without active or past suicidality, and healthy non-suicidal control individuals.

## Methods

### Study population

The population for this study consisted of 264 participants aged 18 years and over, recruited in the area of Padova, Northeast Italy, in the period 2017–2018. Of them, 62 (“cases”) were inpatients hospitalized at the psychiatric ward of the Padova Hospital, Italy, because of an attempted suicide, defined as “A non-habitual act with non-fatal outcome that the individual, expecting, or taking the risk, to die or to inflict bodily harm, initiated and carried out with the purpose of bringing about wanted changes” [23]. The remaining participants belonged to two control groups: a group of inpatients hospitalized at the same Psychiatric ward for reasons other than a recent attempted suicide (*n* = 64; “psychiatric controls”); and a group of 138 patients (“healthy controls”) recruited at their General Practitioner (GP) office. Participants of both control groups were required to have a negative history of suicidal behavior (as self-reported and further confirmed by the GP/medical records), and lack of current suicidal ideation, as indicated by a negative response (“Not at all”) to the fifteenth question (“Thoughts of ending your life”) of the Symptom Check List-90 [24]. Additionally, healthy controls were required to have no history of psychiatric disorders. The only additional inclusion criterion was the ability to correctly read, understand and write in Italian; exclusion criteria were age less than 18 years, mental retardation, severe cognitive impairment and, for all the inpatients, unstable psychiatric conditions.

The study protocol has been approved by the institutional review board (no. 0020095), in accordance with the guidelines of the 1995 Declaration of Helsinki (as revised in Tokyo in 2004). Participation was voluntary, and no fee or other compensation was given for taking part in the study. All participants provided informed consent and all data were collected and stored anonymously.

All inpatients were invited to participate in the study as soon as allowed by their medical conditions, and anyhow within one week after admission. Those who agreed to participate were interviewed by a research team member regarding their sociodemographic (age, sex, education levels, professional status, civil status) and clinical (i.e., psychiatric diagnosis, psychotropic drug use, psychiatric hospitalizations, family mental health history) characteristics; the latter were further confirmed through revision of medical records. In addition, participants were asked to fill-in two self-administered questionnaires to assess Empathy (the Interpersonal Reactivity Index – IRI) [5] and psychopathology (The Symptom Checklist-90 – SCL90) [24]. The questionnaires were completed autonomously by the participants in connection with the interview, under supervision of a research team member. Healthy controls were interviewed during their visit to the GP, and they were invited to autonomously fill-in the IRI questionnaire under supervision of the assessor immediately after their visit to the GP.

All questionnaires were filled under supervision of and returned to the initial assessor, who checked the forms for completeness and correctness, thus minimizing missingness and guaranteeing data quality.

### The questionnaires

Empathy was measured with the validated Italian version of the IRI [25], a self-administered tool developed by Davis [5]. It consists of 28-items spanning the emotional and cognitive components of empathy, with each item scored on a five-point Likert scale (from one, “Does not describe me well”, to five, “Describes me well”). The 28 items are organized into the following four subscales:

1. Perspective Taking (PT): cognitive skills to see things from the point of view of others, the tendency to spontaneously adopt the psychological point of view of others, without necessarily experiencing any affective involvement;
2. Empathic Concern (EC): the ability to feel affective reactions of concern, sympathy, and compassion for other people undergoing negative experiences;
3. Personal Distress (PD): the tendency to feel distress and discomfort in witnessing other people’s suffering and negative experiences;
4. Fantasy (FS): the imaginative ability to transpose oneself and identify strongly with fictitious characters in plays, movies, and books.

In our sample, the Cronbach α for each scale ranged between 0.4 to 0.6, suggesting acceptable reliability.

Levels of psychopathology were measured with the SCL-90, a self-reported questionnaire developed by Derogatis et al. [24], commonly used to evaluate a variety of psychological problems and symptoms of psychopathology. It consists of 90 items scored on a five-point Likert scale; the average of the 90 item scores is measured by the Global Severity Index (GSI), representing the current level of psychopathology. In addition, scores for nine dimensions (somatization, obsessive–compulsive, interpersonal sensitivity, depression, anxiety, hostility, phobic anxiety, paranoid ideation, psychoticism) can be calculated; moreover, seven additional items, mainly focused on appetite and sleep, contribute to the GSI but are not combined in a specific dimension. Reliability in the present sample was excellent, with a Cronbach α of 0.99.

### Statistical analyses

Data were checked for missingness. Only five individual item missing scores were found in the SCL-90 questionnaire; in these cases, the corresponding individual subscale score was computed as the mean of the available subscale items. In case of IRI missing individual item scores (*n* = 6 observations), personal mean imputation of the single item through the personal observed subscale item scores was computed. No other missing observations were detected.

Continuous variables were described via mean and standard deviation, or median and interquartile range, as appropriate; categorical variables were summarized via frequencies and percentages. The differences between the three groups (attempted suicide cases, psychiatric controls, healthy controls) were tested via Χ^2^ test for categorical variables, and via ANOVA or Kruskal–Wallis rank sum test for continuous variables, as appropriate. Post-hoc pairwise comparisons of SCL-90 and IRI scores were performed using the Wilcoxon rank sum test, with adjustment of r multiple testing via the Benjamini–Hochberg method.

Spearman’s rank correlations between IRI empathy dimensions and levels of psychopathology were conducted separately in attempted suicide cases, psychiatric inpatients and healthy controls. The relationships between each IRI subscale, levels of psychopathology (GSI score), and case–control groups were tested via multivariate linear regression models (Model 1), further adjusted for age, sex, civil status, and professional level (Model 2). In an additional model adjusted for covariates, we further tested the interaction between levels of psychopathology and group (Model 3). Supplementary multivariate linear regression models and interaction models were performed to test the associations with distinct dimensions of psychological distress as indicated by the SCL-90. When the interaction term resulted significant in the regression models, group-stratified adjusted analyses (as in Model 2) were conducted.

A *p*-value of < 0.05 was considered significant.

All the analyses were performed with R software version 4.2.3. [26].

## Results

Background characteristics of the 264 patients who participated in the study are reported in Table 1.

**Table 1:** Background characteristics of the 264 participants

	**Cases** (attempted suicide) (*n* = 62)	**Psychiatric controls** (*n* = 64)	**Healthy controls** (*n* = 138)	
Mean (SD); range				***p*-value**
Age, years	49.0 (14.8); 18–86	42.3 (13.9); 18–77	48.7 (15.3); 19–70	0.011
N (%)				
Sex				0.099
F	32 (51.6)	29 (45.3)	84 (60.9)	
M	30 (48.4)	35 (54.7)	54 (39.1)	
Marital status				< 0.001
Single	22 (35.5)	46 (71.9)	47 (34.1)	
Married/in a relationship	24 (38.7)	12 (18.8)	82 (59.4)	
Divorced	11 (17.7)	4 (6.2)	9 (6.5)	
Widowed/other	5 (8.1)	2 (3.1)	0	
Living conditions, alone	18 (29.0)	14 (21.9)	8 (5.8)	< 0.001
Education level				0.228
Low	26 (41.9)	20 (31.2)	38 (27.5)	
Middle	29 (46.8)	31 (48.4)	79 (57.2)	
High	7 (11.3)	13 (20.3)	21 (15.2)	
Professional level				0.019
None	20 (32.3)	33 (51.6)	59 (42.8)	
Low	18 (29.0)	17 (26.6)	20 (14.5)	
Middle	21 (33.9)	11 (17.2)	54 (39.1)	
High	3 (4.8)	3 (4.7)	5 (3.6)	
Main psychiatric diagnosis				< 0.001
Psychotic disorder	7 (11.3%)	24 (37.5%)	–-	
Depressive disorder	40 (64.5%)	12 (18.8%)	–-	
Bipolar disorder	11 (17.8%)	24 (37.5%)		
Other (personality disorder, eating disorder, substance abuse)	4 (6.3%)	4 (6.3%)	–-	
Secondary Diagnosis				< 0.001
Personality disorder	10 (16.1%)	5 (7.8%)	–-	
Substance abuse	3 (4.8%)	3 (4.7%)	–-	
Other	1 (1.6%)	1 (1.6%)	–-	

**Fig. 1:**
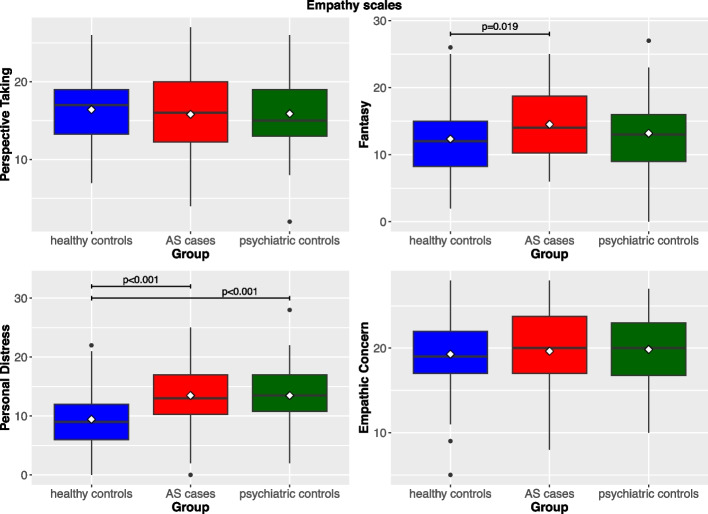
Scores of single Empathy dimensions (IRI scales) in the three study groups. The horizontal line illustrates the median value of GSI score in each group; diamonds are mean values

AS cases were more likely to be divorced or widowed, of low professional level, and to live alone. AS cases and psychiatric controls did not differ in terms of severity of psychopathology at any dimensions, but psychopathology levels were significantly higher in these two groups than in the healthy controls (Additional file 1). Empathy scores for the dimensions of Empathic Concern and Perspective Taking did not differ between the three groups; scores at the dimensions of Fantasy and Personal Distress were significantly higher among cases than in the healthy controls (mean: 14.5 vs. 12.3, SD: 4.7 vs. 5.2, *p* = 0.019; and mean: 13.5 vs. 9.4, SD: 5.3 vs. 9.4, *p* < 0.001, respectively); in addition, Personal Distress levels were significantly higher among psychiatric controls (mean 13.5, SD 5.1) compared to the healthy controls (mean 9.4, SD 5.2, *p* < 0.001) (Fig. 1).

**Fig. 2:**
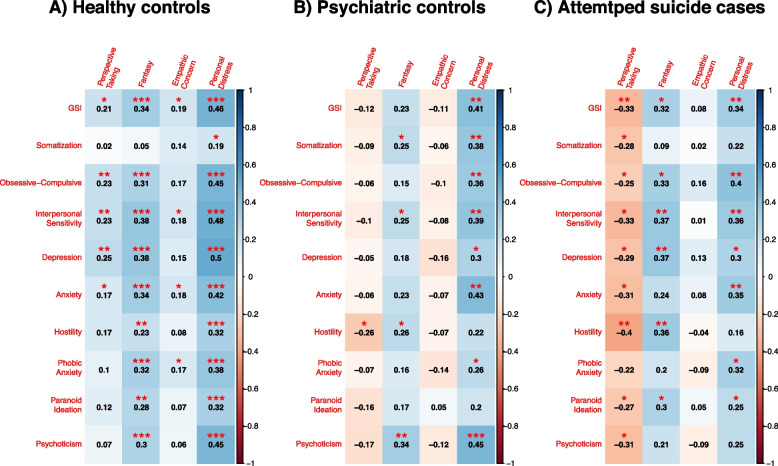
Correlations between Empathy and psychological distress (SCL-90) dimensions in the three groups. Numbers indicate correlation coefficients. Significant correlations are marked with red * (*p < 0.05; ** *p* < 0.01; *** *p* < 0.001)

Correlation analyses showed a number of correlations between levels of psychopathology and scores of the IRI dimensions, with differences in the strength and direction of the correlations between the three groups (Fig. 2). In detail, high levels of psychopathology (GSI score) significantly correlated with higher scores of Personal Distress and Fantasy (*ρ* = 0.34 and *ρ* = 0.32, *p* = 0.007 and *p* = 0.013, respectively), but with lower scores of Perspective Taking (*ρ* = −0.33, *p* = 0.009) among AS cases. Similar, or even stronger correlations between high levels of psychological distress and higher scores of Personal Distress (*ρ* = 0.46, *p* < 0.001), Fantasy (*ρ* = 0.34, *p* < 0.001) and Empathic Concern (*ρ* = 0.19, *p* = 0.029) were found among healthy controls; in addition, in this group, high GSI score significantly correlated with higher levels of Perspective Taking (*ρ* = 0.21, *p* = 0.014). On the contrary, only a significant moderate correlation between high psychopathology levels and high scores of Personal Distress was found among psychiatric controls (*ρ* = 0.41, *p* = 0.001). Similar patterns emerged at all dimensions of psychological distress as assessed by the SCL-90 (Fig. 2). In detail, higher scores of Personal Distress correlated with higher levels of psychological distress at all SCL-90 dimensions in AS cases (except for Somatization, Hostility and Psychoticism), as well as in the psychiatric control group (except for Hostility and Paranoid Ideation); strengths and significance of the correlations were similar in the two groups (*ρ* = 0.25 to 0.4, *p* = 0.001 to 0.046; and *ρ* = 0.26 to 0.45, *p* < 0.001 to 0.036, respectively). In addition, AS cases, but not psychiatric controls, showed consistent patterns of correlations between higher levels of psychopathology and higher Fantasy (*ρ* = 0.3 to 0.37, *p* = 0.003 to 0.016; except for Somatization, Anxiety, Phobic Anxiety and Psychoticism), but lower Perspective Taking (*ρ* = *-*0.25 to −0.4, *p* = 0.001 to 0.047; except for Phobic Anxiety). The healthy control group displayed highly consistent patterns of correlations between higher levels of psychological distress and higher levels of Personal Distress (*ρ* = 0.19 to 0.5, *p* < 0.001 to 0.027), Fantasy (except for Somatization; *ρ* = 0.23 to 0.38, *p* < 0.001 to 0.006) and, to a lesser extent, Perspective Taking (except for Somatization, Hostility, Phobic Anxiety, Paranoid Ideation and Psychoticism; *ρ* = 0.17 to 0.25, *p* = 0.003 to 0.047).

**Fig. 3:**
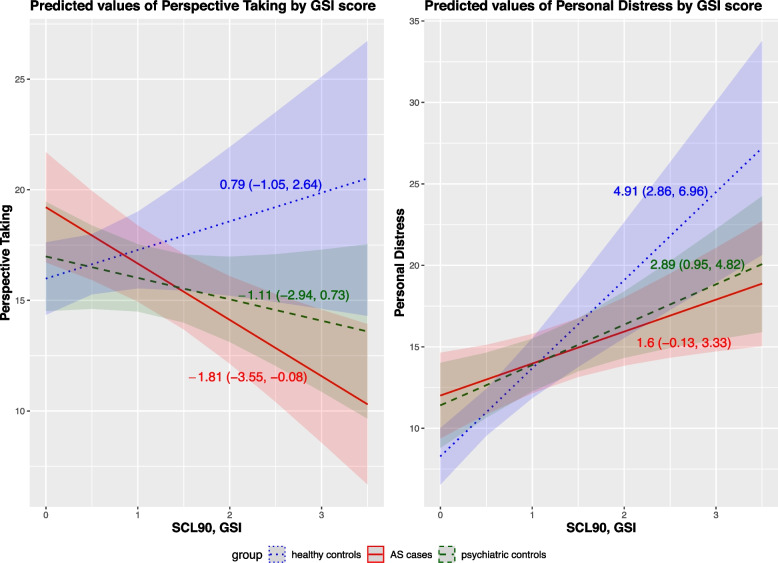
Predicted values of Perspective Taking (dependent variable; left-hand side) and Personal Distress (dependent variable; right-hand side) by psychological distress level (GSI score, main predictor) in the three groups. Results are from multivariate linear regression models adjusted for age, sex, civil status and professional level. Numbers are unstandardized regression coefficients with 95% confidence intervals

In linear regression analyses, higher scores of psychological distress (GSI score) were associated with higher scores at empathy dimensions of Fantasy (B = 2.78, 95% CI = 1.74 to 3.82, *p* < 0.001) and Personal Distress (B = 3.47, 95% CI = 2.48 to 4.46, *p* < 0.001), irrespective of the group (Model 1), even after controlling for background characteristics (age, sex, civil status, and professional levels) (Model 2) (B = 2.10, 95% CI = 1.08 to 3.13, *p* = 0.0001; and B = 2.90, 95% CI = 1.87 to 3.93, *p* < 0.001, respectively). In addition, the adjusted model revealed a negative relation of the empathy dimension of Perspective Taking with psychological distress (B = −1.15, 95% CI = −2.14 to −0.17, *p* = 0.021) (Table 2). Higher age and male sex were also associated with lower Perspective Taking and Fantasy scores, and with lower Personal Distress score, respectively. The associations between GSI and empathy scale scores remained unchanged in sex-stratified analyses, with the exception of Perspective Taking, which was not significant in either group (results not shown). No significant associations emerged between empathy dimensions and the case/control status. However, the case/control group interacted with the GSI score in explaining the empathic dimensions of Perspective Taking and Personal Distress (Table 2, Fig. 3).

**Table 2:** Associations between levels pf psychopathology and case-control group vs. Empathy scores

	**Model 1**		**Model 2**		**Model 3**	
	**B**	**95% CI**	**B**	**95% CI**	**B**	**95% CI**
Empathy: Perspective Taking						
GSI	-0.70	-1.66 to 0.26	*-1.15*	*-2.14 to -0.17*	1.29	-0.69 to 3.28
Group						
Attempted suicide cases	-0.01	-1.61 to 1.62	0.57	-1.10 to 2.25	*3.23*	*0.84 to 5.61*
Psychiatric controls	0.01	-1.54 to 1.56	0.17	-1.48 to 1.81	1.00	-1.46 to 3.47
GSI x Attempted suicide cases	---	---	---	---	*-3.84*	*-6.26 to -1.42*
GSI x Psychiatric controls	---	---	---	---	-2.26	-4.78 to 0.25
Empathy: Fantasy						
GSI	*2.78*	*1.74 to 3.82*	*2.10*	*1.08 to 3.13*	*3.21*	*1.11 to 5.32*
Group						
Attempted suicide cases	-0.26	-2.01 to 1.49	0.40	-1.35 to 2.15	1.39	-1.14 to 3.92
Psychiatric controls	-1.28	-2.96 to 0.40	-1.54	-3.25 to 0.18	-0.92	-3.53 to 1.70
GSI x Attempted suicide cases	---	---	---	---	-1.57	-4.13 to 1.00
GSI x Psychiatric controls	---	---	---	---	-1.24	-3.91 to 1.43
Empathy: Empathic Concern						
GSI	0.13	-0.78 to 1.04	-0.10	-1.06 to 0.85	0.58	-1.38 to 2.53
Group						
Attempted suicide cases	0.25	-1.29 to 1.78	0.61	-1.02 to 2.23	0.83	-1.53 to 3.18
Psychiatric controls	0.46	-1.01 to 1.93	0.85	-0.75 to 2.44	1.67	-0.76 to 4.10
GSI x Attempted suicide cases	---	---	---	---	-0.65	-3.03 to 1.73
GSI x Psychiatric controls	---	---	---	---	-1.15	-3.63 to 1.32
Empathy: Personal Distress						
GSI	*3.47*	*2.48 to 4.46*	*2.90*	*1.87 to 3.93*	*5.41*	*3.31 to 7.50*
Group						
Attempted suicide cases	1.02	-0.65 to 2.69	1.62	-0.14 to 3.38	*3.73*	*1.21 to 6.26*
Psychiatric controls	1.38	-0.22 to 2.98	1.58	-0.15 to 3.31	*3.13*	*0.53 to 5.73*
GSI x Attempted suicide cases	---	---	---	---	*-3.45*	*-6.00 to -0.89*
GSI x Psychiatric controls	---	---	---	---	*-2.93*	*-5.59 to -0.28*

Specifically, the results of Table 2, model 3, suggest a significant interaction among GSI and the case/control group for PD, and a statistically non-significant interaction for controls in relation to PT. Overall, results indicate that GSI had a negative effect on PT in the population, while including an interaction we observed a positive effect of GSI for AS patients (1.29 + 3.23–3.84 = 0.71) only. In group-stratified analyses, with increasing levels of psychopathology (GSI), scores of Perspective Taking decreased steeply and significantly in individuals who had recently attempted suicide (B = −1.81, 95% CI = −3.55 to −0.08, *p* = 0.041), but only tended to decrease in non-suicidal psychiatric inpatients (B = −1.11, 95% CI = −2.94 to 0.73, *p* = 0.237); conversely, scores of Perspective Taking increased with increasing levels of psychological distress in healthy controls (B = 0.79, 95% CI = −1.05 to 2.64, *p* = 0.399). Scores of Personal Distress increased significantly with increasing levels of psychopathology in the control groups (psychiatric inpatients: B = 2.89, 95% CI = 0.95 to 4.82, *p* = 0.004), the steepest slope being among healthy controls (B = 4.91, 95% CI = 2.86 to 6.96, *p* < 0.001), but clearly less steeply and not significantly among AS cases (B = 1.6, 95% CI = −0.13 to 3.33, *p* = 0.070) (Fig. 3). However, in all the stratified analyses, CIs were largely overlapped, indicating a lack of statistically significant distinction between the three groups.

Analyses of specific dimensions of psychopathology are illustrated in figures in Additional file 2, Additional file 3, Additional file 4 and Additional file 5. Irrespective of the group, higher levels of psychopathology at all dimensions were related to higher scores of Personal Distress and Fantasy (B = 1.24 to 3.28, *p* < 0.001 to 0.007; and B = 0.94 to 2.21, *p* < 0.001 to 0.040, respectively). Conversely, lower levels of psychopathology at many dimensions (Somatization, Hostility, Phobic Anxiety, Paranoid Ideation, and Psychoticism) were associated with higher levels of Perspective Taking (B = −0.44 to −1.53, *p* < 0.001 to 0.048). On the other hand, irrespective of psychopathology dimension levels, being a psychiatric inpatient and, to a lesser extent, an AS case, were associated with higher scores of Personal Distress compared to the healthy controls; AS cases also had a tendency for higher scores of Fantasy. No or marginal associations were found between psychopathology levels and scores of Empathic Concern.

Interaction analyses revealed a consistent pattern of associations between levels of psychopathology scales and empathy, which tended to differ between the groups, although not reaching statistical significance (figures in Additional file 6, Additional file 7, Additional file 8 and Additional file 9). Specifically, with increasing levels of psychological distress at most dimensions (Obsessive–compulsive, Interpersonal Sensitivity, Depression, Anxiety, Hostility, Paranoid Ideation, Psychoticism), scores of Perspective Taking decreased or tended to decrease in AS cases, tended to increase in healthy controls, and remained almost unchanged in psychiatric controls. On the other hand, with increasing levels of psychopathology (Depression, Anxiety, Interpersonal Sensitivity, Hostility, Phobic Anxiety and Psychoticism), scores of Personal Distress increased marginally in AS cases and psychiatric controls, but steeply in healthy controls. However, in all these stratified analyses, the CIs were largely overlapped, again indicating a lack of statistically significant distinction between the three groups.

## Discussion

The main finding of this study is that scores at different dimensions of empathy are related primarily to levels of psychological distress or psychopathology, rather than to a status of having recently attempted suicide or being a non-suicidal psychiatric patient. However, the pattern of relation between psychological distress and empathy appeared to vary between the groups, with a much more dampened association in psychiatric patients and even more in individuals who have recently attempted suicide. Conversely, individuals with no psychiatric conditions appeared to manifest a much more enhanced relation between empathy and psychological distress.

Empathy is characterized by multiple dimensions fairly different from each other, distinguished into more emotional vs. more cognitive, and self-oriented vs. other-oriented [3, 4]. As such, the different empathy dimensions deserve to be analyzed separately. Personal Distress and Perspective Taking are poles apart, as confirmed by results of our study, with higher levels of psychopathology being related to higher scores of Personal Distress, but to lower scores of Perspective Taking.

Based on our finding of a tendency for stronger relations between psychopathology/psychological distress and empathy in healthy controls than in the other groups, it is tempting to speculate that even low levels of psychological distress may elicit an “empathic reaction” in healthy individuals. In these individuals, the reaction appears to be both hetero-oriented, with increased levels of Perspective Taking, i.e. a tendency to better understand others’ pain, and self-oriented, with higher levels of Personal Distress. It seems that healthy individuals respond to a stressful situation by trying to better understand others’ pain and to improve their own capacity to understand others. At the same time, a situation of psychological distress may elicit a strong feeling of personal distress, making these individuals more “sensitive” to feeling others’ pain, probably as a consequence of not being used to experiencing such psychological distress. In other words, first time, or “non-habitual” experience of psychological distress may induce an acute stress reaction. This is in line with results of a Japanese study conducted on a group of disaster workers, showing an association between high levels of Personal Distress (and partially of Fantasy) with post-traumatic stress responses and general psychological distress [7]. Our results are also in line with those of several works showing enhanced prosocial behavior and/or emotional, but not cognitive empathy after a psychosocial stressor in healthy individuals [27].

On the other hand, it seems that individuals who have been suffering from more severe psychological distress somehow develop a “habituation” to levels of distress, which consequently does not elicit any strong empathic reaction. Partly related to this observation are the results of a meta-analysis on the Interpersonal Reactivity Index in schizophrenia patients [9], showing that those with a longer duration of illness exhibited greater deficits in perspective taking; however, no effects of duration of illness were found for personal distress or fantasy levels. This appears to be even truer among individuals who have recently attempted suicide, who somehow lose their ability to understand others’ pain, but also tend to personally feel less intensively the others’ pain. Based on our results, this trait seems to distinguish suicidal from non-suicidal patients, with suicidal individuals being more detached from what happens around them. Rather, the higher their levels of psychological distress, the lower their ability to understand others' pain, as if they were focused on their own suffering. However, it must be noted that empathy profiles did not significantly differ between psychiatric controls and AS cases, while both these groups had higher levels of PD compared to healthy individuals. Interestingly, the results were rather consistent across different dimensions of psychopathology. This finding is in line with those of a previous study showing that PD was the strongest predictor of personality organization, and in particular of poor levels of personality organization and functioning, among psychiatric inpatients, but none of the empathic subscales was a good predictor of the categorical psychiatric diagnosis [28]. On this regard, it is likely that comorbid conditions, such as personality disorders, contribute to shape empathy profiles particularly in individuals suffering from psychiatric disorders. For example, borderline personality disorder is characterized by impaired social functioning and empathy anomaly [29], with empathy varying depending on comorbid psychiatric conditions and perceived stress [27]. In particular, patients with borderline personality disorder have lower emotional, but not cognitive empathy in reaction to a stressor than healthy controls, although in baseline conditions they have been repeatedly found with higher PD than healthy ones [30]. The authors hypothesize that healthy individuals can adopt social skills to buffer stress effects, a skill which is missing in patients with borderline personality disorder [27]. However, while personality disorders and traits may in fact influence individual empathy attitude, it should be noticed that our study did employ a dimensional approach to the study of psychopathology and its relationship with empathy. Of note, the SCL-90 used to measure psychological distress in our study, and its interpersonal subscales in particular (Interpersonal Sensitivity, Hostility, Paranoid Ideation) capture dimensions such as interpersonal dysfunction typical of many personality disorders [31].

It must be noticed that, since this is a cross-sectional study, it is not possible to identify a cause-and-effect relationship between the detected associations. In other words, it is possible that an empathic attitude characterized by high levels of PD induces higher psychological distress in healthy individuals, but much less distress in psychiatric patients, and especially in AS cases. Another possible explanation to our findings could be related to the observation that, as suggested by Kaluzeviciute [32], psychiatric patients are often not able to recognize and properly respond to others’ emotions, this process characterizing their dysfunctional nuclei. Furthermore, given that empathy is a crucial human ability in the context of prosocial behavior and moral development, it is likewise plausible that impaired empathy contributes to the development of a psychopathologic condition. Tone and Tully [33] hypothesized a model in which typical development of affective and cognitive empathy can be influenced by complex interplay among intraindividual and interindividual moderators that increase risk for empathic personal distress and excessive interpersonal guilt. Learning to respond to others’ distress with well-regulated empathy is an important developmental task linked to positive health outcomes and moral achievements. However, this important interpersonal skill set may also, paradoxically, confer risk for depression and anxiety when present at extreme levels and in combination with certain individual characteristics or within particular contexts.

The relationship between empathy and the level of psychopathology is therefore complex and articulated. Thus, the findings of this study should be interpreted considering some limitations. First, the sample size was relatively small; hence, some of the associations that were detected only as trends could have become significant with a greater number of subjects recruited. This is especially true for subgroup analyses, where the small sample size may have importantly impacted statistical power, thus limiting the ability to detect differences. Thus, results of subgroup comparisons should be interpreted with caution. Furthermore, the cross-sectional study design is weak for testing causality. Data collection consistency was not checked via, e.g., inter-rater reliability. However, data were collected through interviews, and self-reported questionnaires were filled in under supervision of an experienced assessor, who checked the filled forms for clarity and completeness, this ensuring data quality. Reliability of the empathy subscales in our study was rather low (Cronbach α of 0.4–0.6), this possibly negatively impacting the robustness of the results. While we cannot rule out that the modest internal consistency of the IRI subscales may have biased our results, in particular the specificity of the associations with certain empathic dimensions, the consistently opposite directions of the associations between self-oriented vs. other-oriented empathy dimensions and psychological distress support the validity of our findings. In addition, the two tools used to assess the key measures of this study (i.e., the IRI and the SCL-90) are widely used, well-validated self-reported instruments, and the IRI has shown adequate validity and reliability in different settings and populations [34]. Moreover, although the IRI is a self-reported instrument, and as such has some limitations (e.g., response and presentation bias), more objective behavioral or neuroscientific measures are expensive and still lacking confirmation in terms of reliability and validity [35]. In addition, the study was conducted across a two-year period between 2017 and 2018; however, we believe that our data and results are still valid, given the reliability of the instruments employed in the assessments. Lastly, the absence of data on disease duration prevented us to evaluate how and if they moderated the association between levels of empathy and psychopathology in patients who had attempted suicide and in psychiatric controls.

Even with the above limitations, our findings have important clinical implications for the development of preventive and therapeutic interventions aimed at reducing suicide risk. In particular, our findings of self-oriented empathy being related to psychological distress could represent an important tool to inform interventions or screening strategies in psychiatric settings. Given that empathy dimensions, particularly Perspective Taking and Personal Distress, are strongly associated with psychological distress and psychopathology, interventions targeting these specific aspects could be valuable in mitigating suicide risk in psychiatric populations. For instance, enhancing Perspective Taking—the cognitive ability to understand and adopt another person’s viewpoint—could be an effective therapeutic strategy. Cognitive-based interventions, such as Cognitive Behavioral Therapy (CBT) and Mentalization-Based Therapy (MBT), have been shown to improve perspective-taking abilities in individuals with psychiatric conditions [36, 37]. By fostering a greater understanding of others’ emotions and experiences, individuals at risk for suicide may develop stronger interpersonal connections, reducing feelings of isolation and hopelessness, which are key risk factors for suicidal behavior. Moreover, interventions that incorporate role-playing exercises, perspective-shifting tasks, and social cognition training could further enhance this skill and promote emotional regulation in high-risk populations. At the same time, mitigating Personal Distress—which refers to the self-oriented emotional response to others' suffering—may help individuals develop greater emotional resilience and reduce their susceptibility to overwhelming distress. Emotion regulation strategies, such as Dialectical Behavior Therapy (DBT), which is particularly effective in populations with heightened emotional sensitivity (e.g., individuals with borderline personality disorder) [38], could be employed to help individuals to manage excessive self-focused distress. Mindfulness-based interventions (MBIs), such as Mindfulness-Based Stress Reduction (MBSR), may also be beneficial in decreasing maladaptive Personal Distress responses by helping individuals to develop greater self-awareness and emotional regulation skills without becoming emotionally overwhelmed by others’ suffering [39]. Additionally, group-based interventions could serve a dual function in both enhancing Perspective Taking and reducing Personal Distress by encouraging individuals to share experiences and develop a sense of social belonging. Compassion-Focused Therapy (CFT), which emphasizes self-compassion and the ability to extend compassion toward others, could be particularly useful for individuals who experience high Personal Distress in response to social and emotional cues [40]. These targeted interventions could be particularly relevant in psychiatric inpatient settings, where patients may experience chronic psychological distress but lack adequate social cognitive skills to regulate their empathic responses. Future studies should explore how these therapeutic strategies can be integrated into clinical practice, potentially leading to more personalized and effective suicide prevention programs tailored to the unique empathy profiles of psychiatric populations.

## Conclusions

In conclusion, it seems that empathy is better described by the level of psychopathology rather than by the status of having attempted suicide. High levels of self-oriented empathy are more characteristic of individuals with a history of suicide attempt, and with high levels of psychopathology. The relationship between psychological distress and empathy seems more dampened in psychiatric patients and in individuals who have recently attempted suicide, but more enhanced in individuals with no psychiatric conditions. Further studies are needed to understand whether emotional and self-oriented dimensions of empathy characterize the suicidal population and could contribute to the identification of people at risk of suicidal behavior.

## Supplementary Information

Additional file 1. Levels of psychopathology/psychological distress in the three study groups. The horizontal line illustrates the median value of GSI score in each group; diamonds are mean values.

Additional file 2. Associations between Empathy dimension of Perspective Taking (dependent variable), single dimensions of psychological distress (SCL-90) and group status (main predictors). Results are from multivariate linear regression models adjusted for age, sex, civil status and professional level.

Additional file 3. Associations between Empathy dimension of Fantasy (dependent variable), single dimensions of psychological distress (SCL-90) and group status (main predictors). Results are from multivariate linear regression models adjusted for age, sex, civil status and professional level.

Additional file 4. Associations between Empathy dimension of Personal Distress (dependent variable), single dimensions of psychological distress (SCL-90) and group status (main predictors). Results are from multivariate linear regression models adjusted for age, sex, civil status and professional level.

Additional file 5. Associations between Empathy dimension of Empathic Concern (dependent variable), single dimensions of psychological distress (SCL-90) and group status (main predictors). Results are from multivariate linear regression models adjusted for age, sex, civil status and professional level.

Additional file 6. Predicted values of Perspective Taking (dependent variable) by SCL-90 psychological distress dimensions of Somatization, Obsessive-Compulsive, Paranoid Ideation and Psychoticism (main predictors) in AS cases, psychiatric controls and healthy controls. Results are from multivariate linear regression models adjusted for age, sex, civil status and professional level; each SCL-90 dimension was entered in separate regression models as main predictors. Numbers are unstandardized regression coefficients with 95% confidence intervals.

Additional file 7. Predicted values of Perspective Taking (dependent variable) by SCL-90 psychological distress dimensions of Interpersonal Sensitivity, Depression, Anxiety and Hostility (main predictors) in AS cases, psychiatric controls and healthy controls. Results are from multivariate linear regression models adjusted for age, sex, civil status and professional level; each SCL-90 dimension was entered in separate regression models as main predictors. Numbers are unstandardized regression coefficients with 95% confidence intervals.

Additional file 8. Predicted values of Personal Distress (dependent variable) by SCL-90 psychological distress dimensions of Interpersonal Sensitivity, Depression, Anxiety and Hostility (main predictors) in AS cases, psychiatric controls and healthy controls. Results are from multivariate linear regression models adjusted for age, sex, civil status and professional level; each SCL-90 dimension was entered in separate regression models as main predictors. Numbers are unstandardized regression coefficients with 95% confidence intervals.

Additional file 9. Predicted values of Personal Distress (dependent variable) by SCL-90 psychological distress dimensions of Phobic Anxiety and Psychoticism (main predictors) in AS cases, psychiatric controls and healthy controls. Results are from multivariate linear regression models adjusted for age, sex, civil status and professional level; each SCL-90 dimension was entered in separate regression models as main predictors. Numbers are unstandardized regression coefficients with 95% confidence intervals.

## Data Availability

The data that support the findings of this study are not openly available due to reasons of sensitivity and are available from the corresponding author upon reasonable request.

## Abbreviations

AS: Attempted Suicide
CI: Confidence Interval
EC: Empathic Concern
FS: Fantasy
GSI: Global Severity Index
IRI: Interpersonal Reactivity Index
PD: Personal Distress
PT: Perspective Taking
SCL90: Symptom Checklist-90
SD: Standard Deviation
