# Supplementary figures and images for: Empathy, psychopathology and suicidal behavior: a case–control study
Source: BMC Psychiatry. 2025 Aug 26;25:811. doi: 10.1186/s12888-025-07230-3 (PMC12379465; doi:10.1186/s12888-025-07230-3)

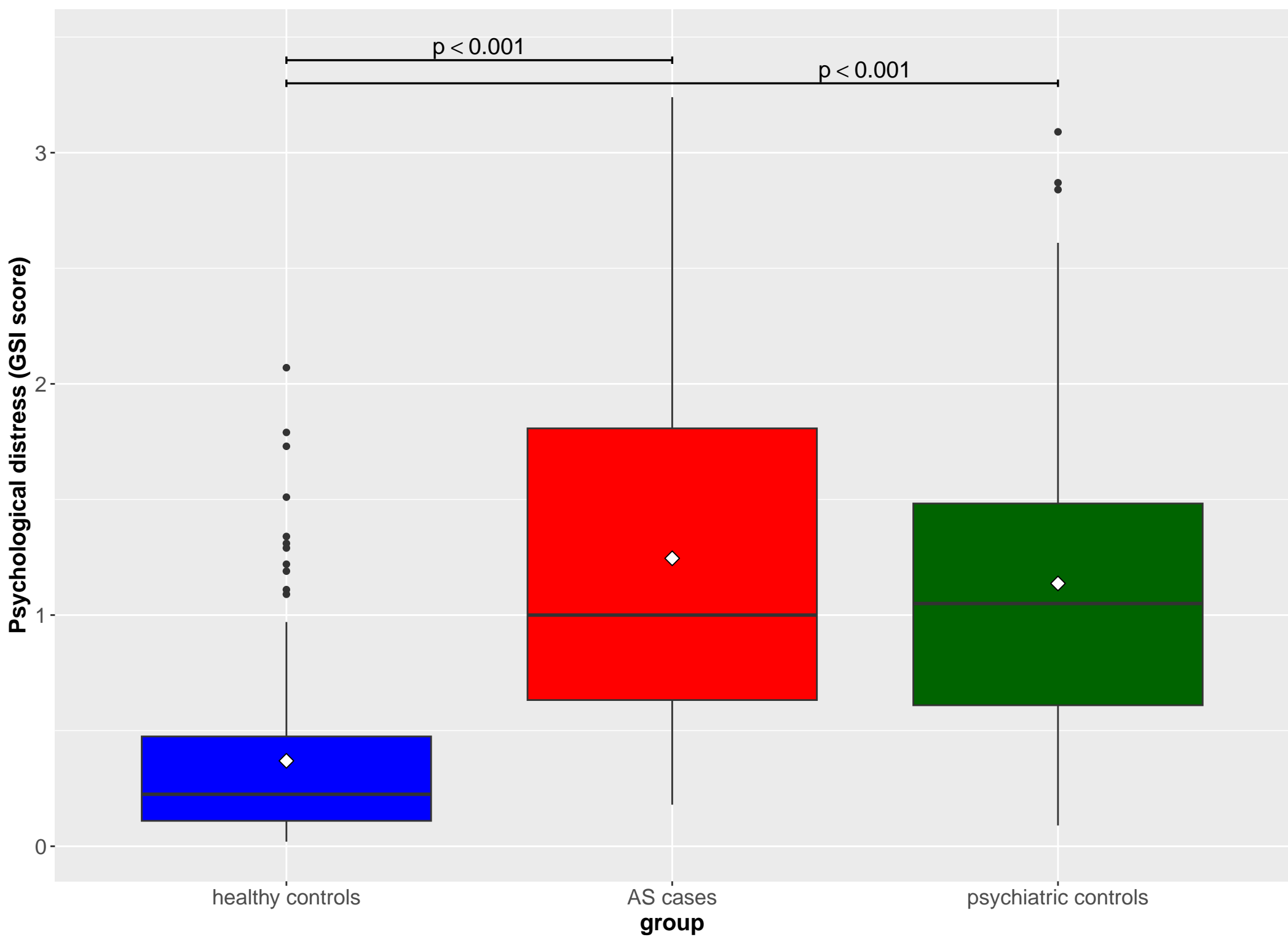

Supplement: Supplementary file 1 — Additional file 1. Levels of psychopathology/psychological distress in the three study groups. The horizontal line illustrates the median value of GSI score in each group; diamonds are mean values. [file 12888_2025_7230_MOESM1_ESM.pdf]

# Fantasy

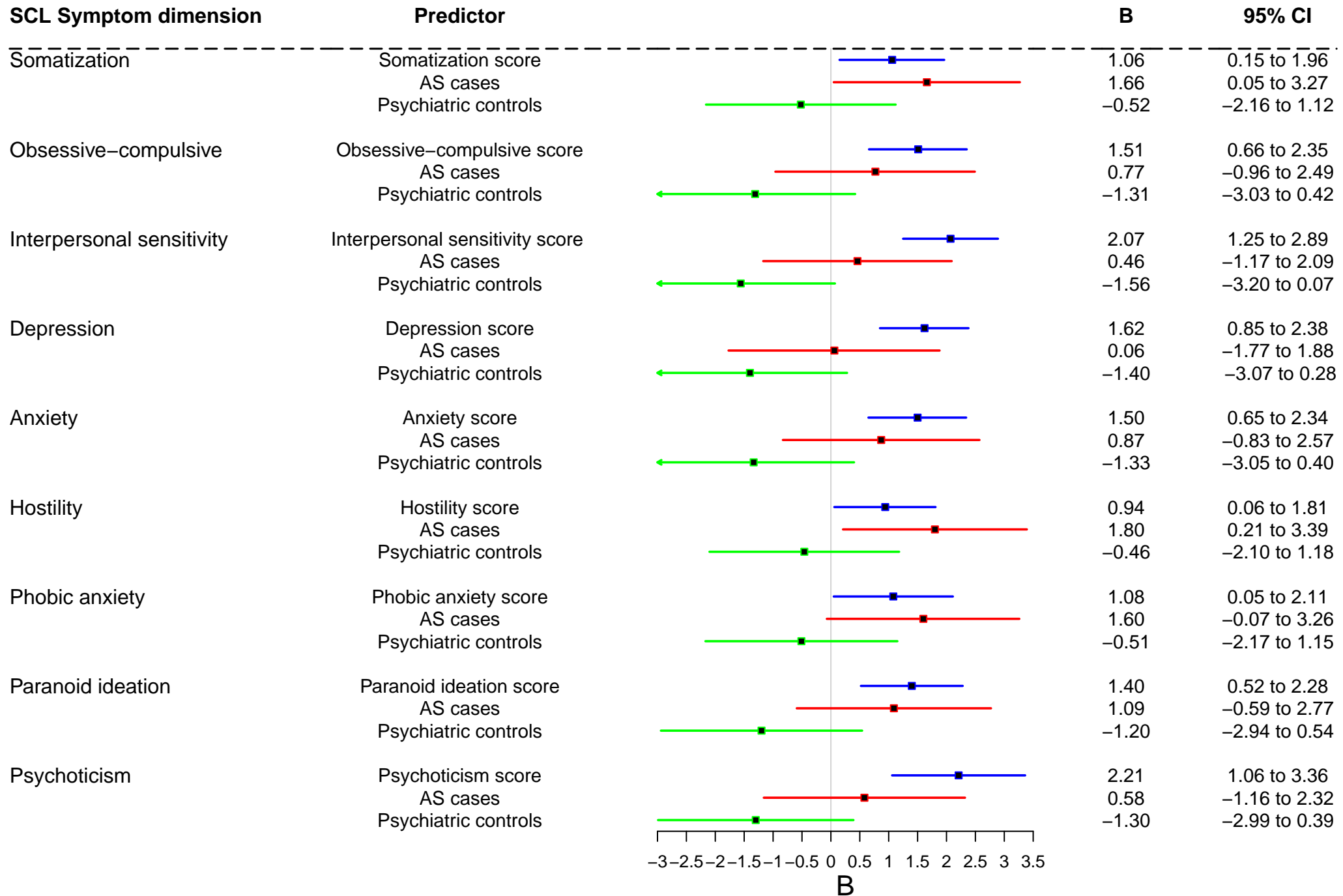

Supplement: Supplementary file 2 — Additional file 2. Associations between Empathy dimension of Perspective Taking (dependent variable), single dimensions of psychological distress (SCL-90) and group status (main predictors). Results are from multivariate linear regression models adjusted for age, sex, civil status and professional level. [file 12888_2025_7230_MOESM2_ESM.pdf]

## Personal Distress

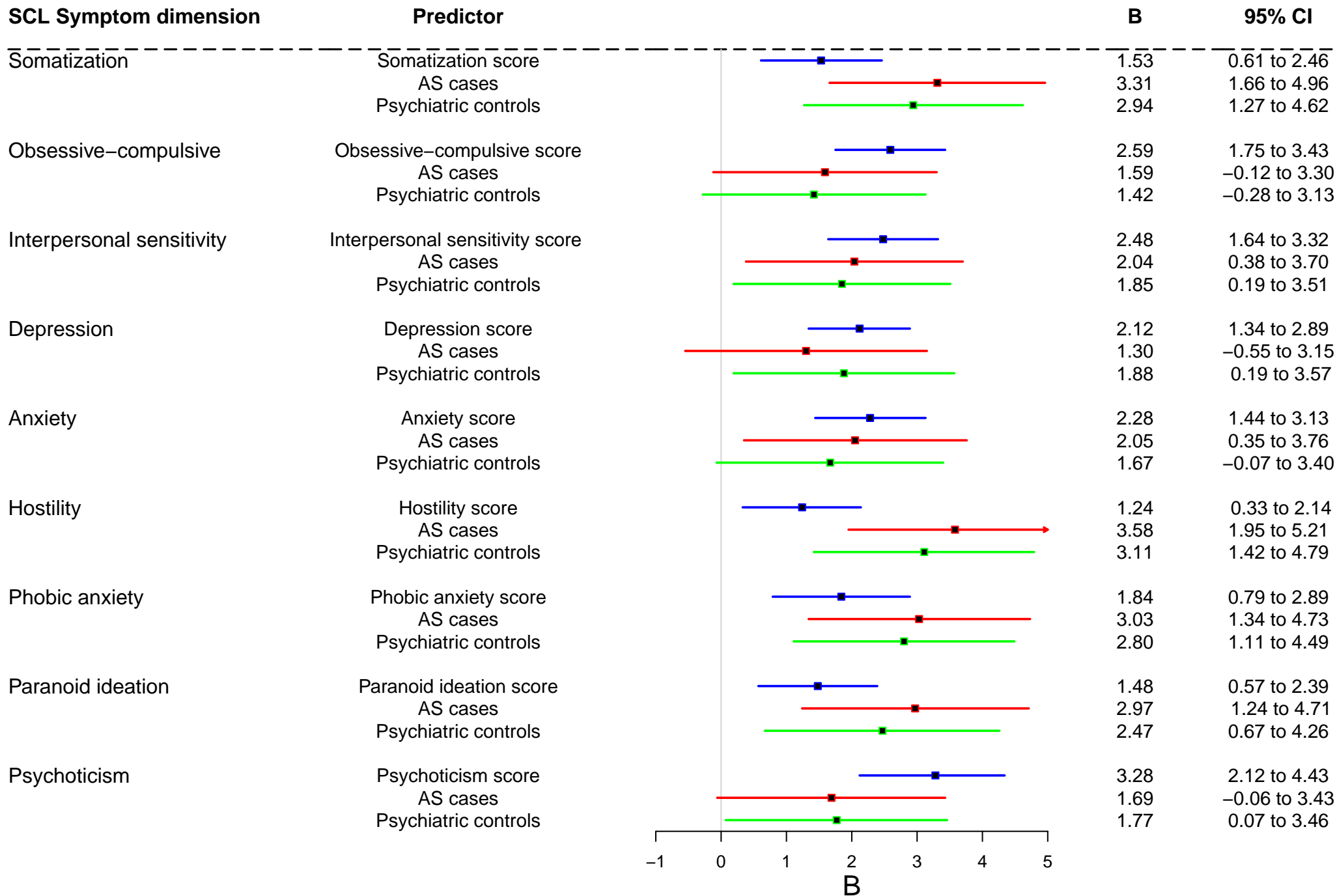

Supplement: Supplementary file 3 — Additional file 3. Associations between Empathy dimension of Fantasy (dependent variable), single dimensions of psychological distress (SCL-90) and group status (main predictors). Results are from multivariate linear regression models adjusted for age, sex, civil status and professional level. [file 12888_2025_7230_MOESM3_ESM.pdf]

## Perspective Taking

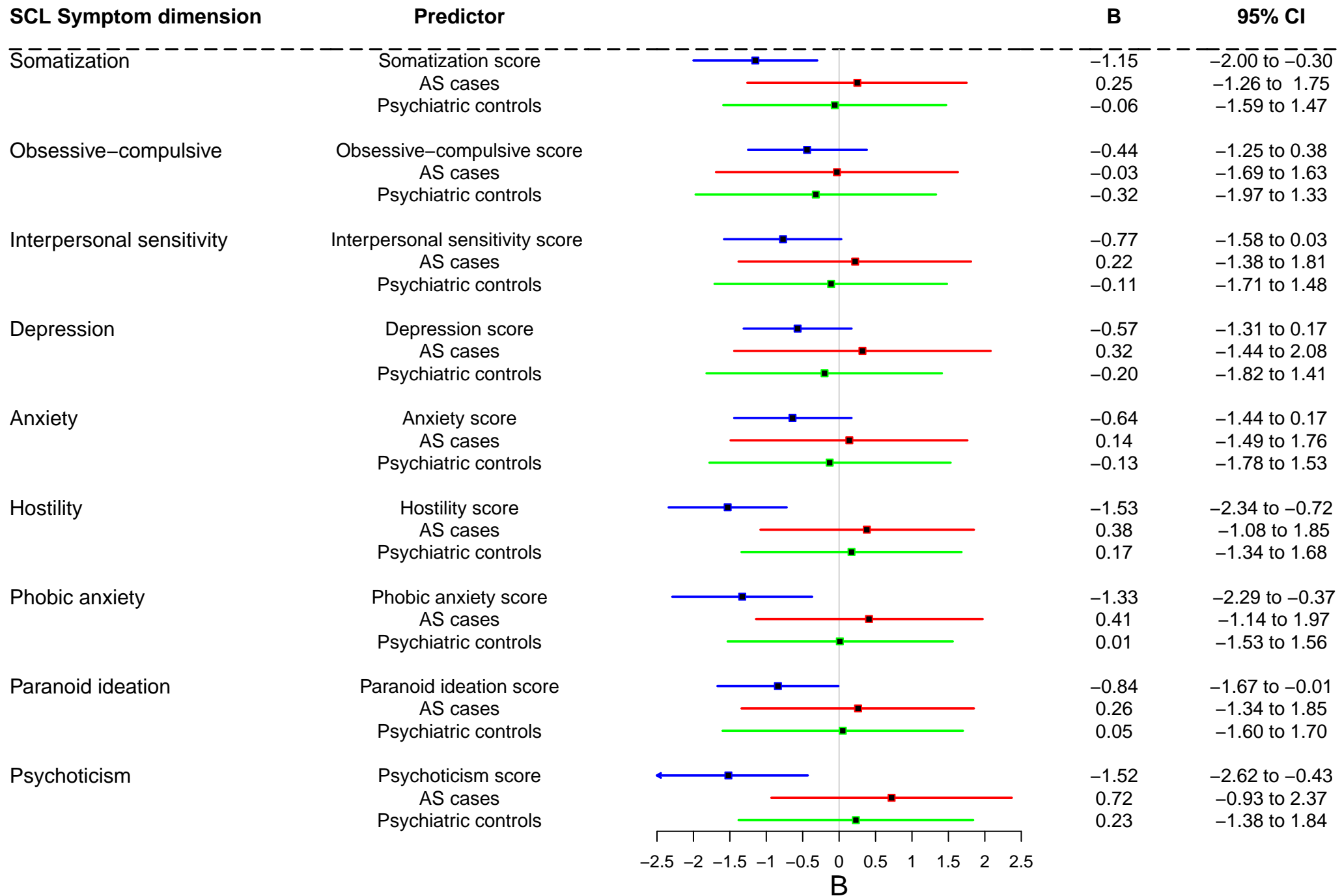

Supplement: Supplementary file 4 — Additional file 4. Associations between Empathy dimension of Personal Distress (dependent variable), single dimensions of psychological distress (SCL-90) and group status (main predictors). Results are from multivariate linear regression models adjusted for age, sex, civil status and professional level. [file 12888_2025_7230_MOESM4_ESM.pdf]

## Empathic concern

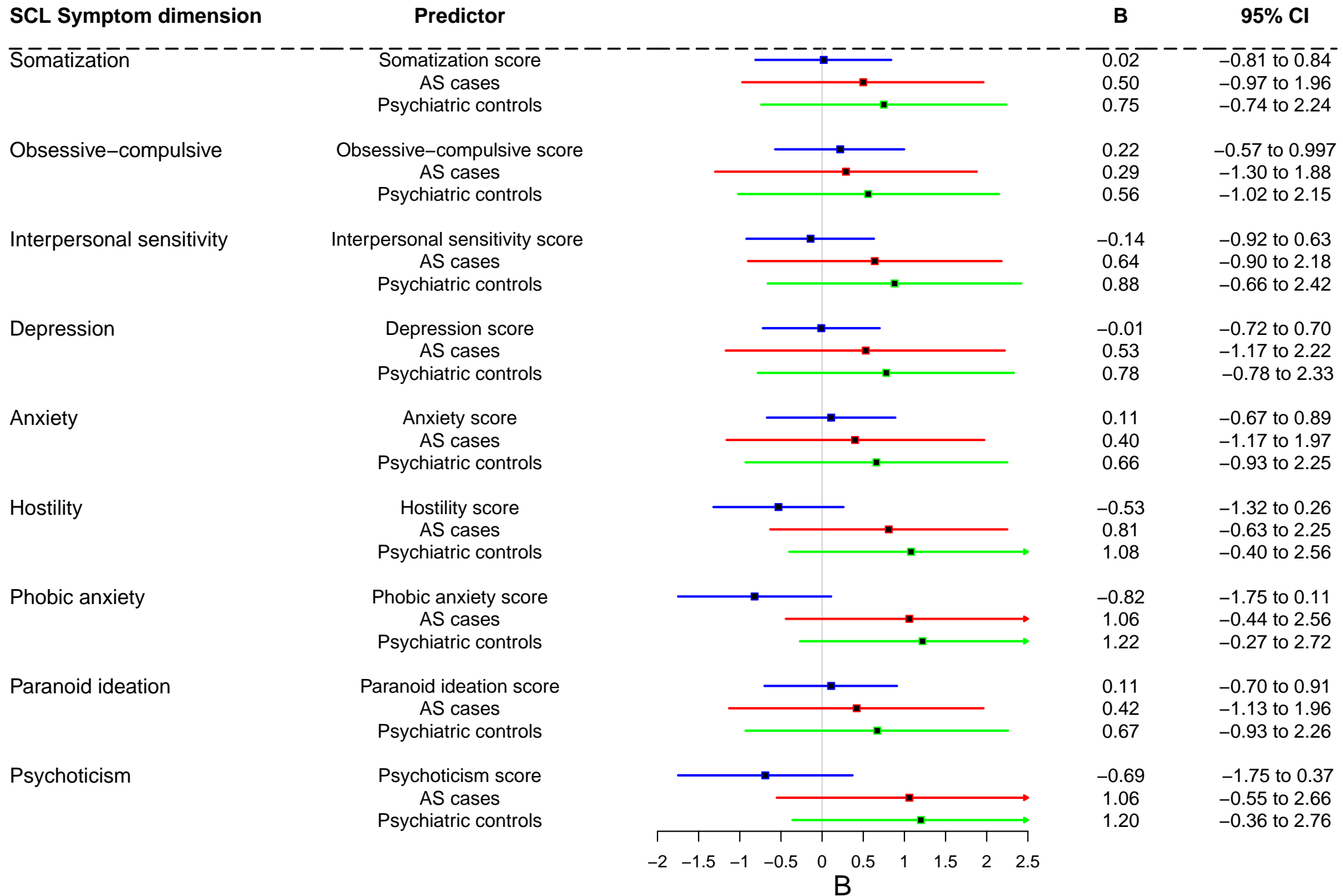

Supplement: Supplementary file 5 — Additional file 5. Associations between Empathy dimension of Empathic Concern (dependent variable), single dimensions of psychological distress (SCL-90) and group status (main predictors). Results are from multivariate linear regression models adjusted for age, sex, civil status and professional level. [file 12888_2025_7230_MOESM5_ESM.pdf]
