# Supplementary material for: Empathy, psychopathology and suicidal behavior: a case–control study
Source: BMC Psychiatry. 2025 Aug 26;25:811. doi: 10.1186/s12888-025-07230-3 (PMC12379465; doi:10.1186/s12888-025-07230-3)
Supplement: Supplementary file 6 — Additional file 6. Predicted values of Perspective Taking (dependent variable) by SCL-90 psychological distress dimensions of Somatization, Obsessive-Compulsive, Paranoid Ideation and Psychoticism (main predictors) in AS cases, psychiatric controls and healthy controls. Results are from multivariate linear regression models adjusted for age, sex, civil status and professional level; each SCL-90 dimension was entered in separate regression models as main predictors. Numbers are unstandardized regression coefficients with 95% confidence intervals. [file 12888_2025_7230_MOESM6_ESM.pdf]

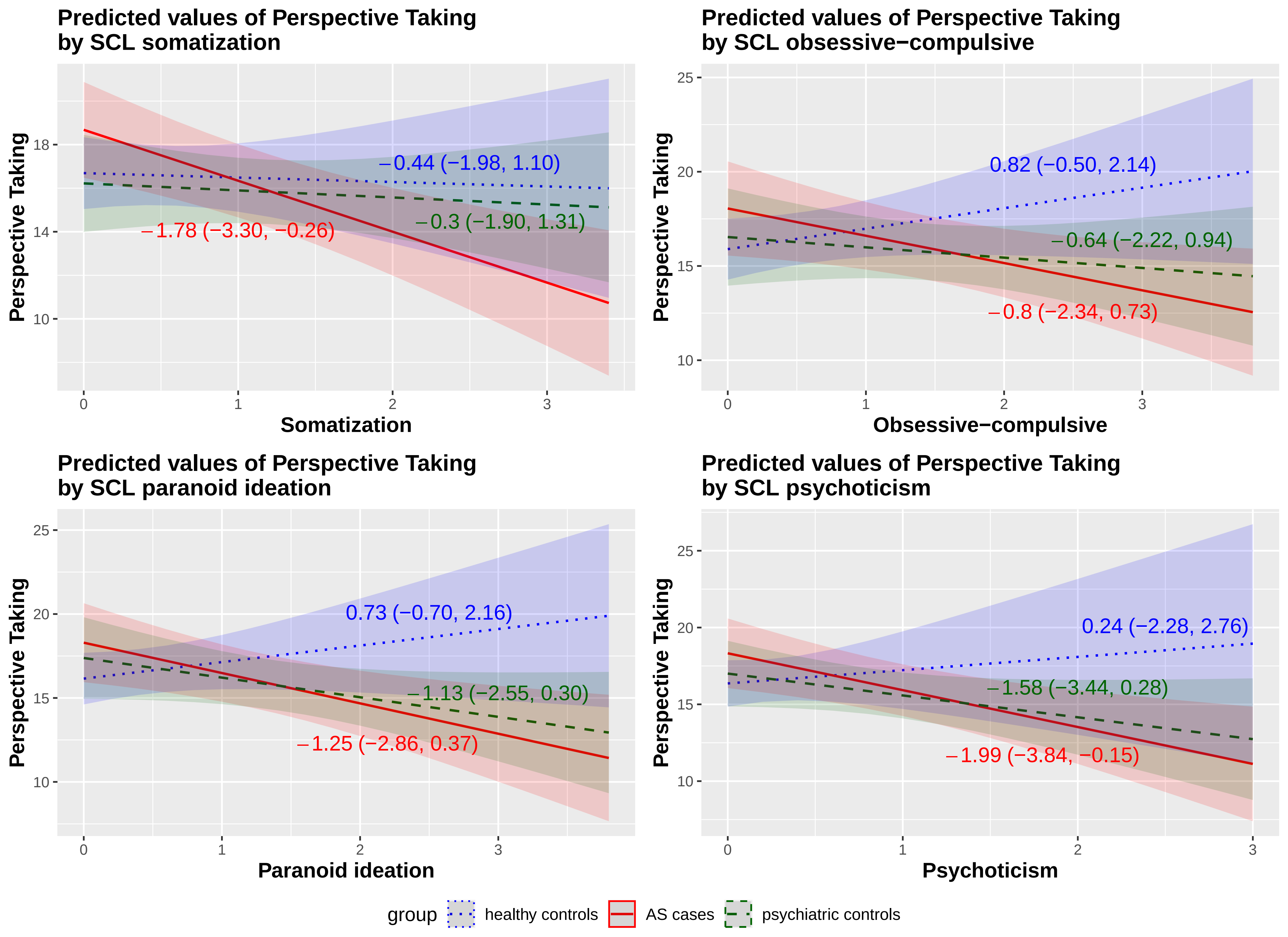

Predicted values of Perspective Taking by SCL somatization
Perspective Taking
Somatization
−0.44 (−1.98, 1.10)
−0.3 (−1.90, 1.31)
−1.78 (−3.30, −0.26)
Predicted values of Perspective Taking by SCL obsessive−compulsive
Obsessive−compulsive
0.82 (−0.50, 2.14)
−0.64 (−2.22, 0.94)
−0.8 (−2.34, 0.73)
Predicted values of Perspective Taking by SCL paranoid ideation
Paranoid ideation
0.73 (−0.70, 2.16)
−1.13 (−2.55, 0.30)
−1.25 (−2.86, 0.37)
Predicted values of Perspective Taking by SCL psychoticism
Psychoticism
0.24 (−2.28, 2.76)
−1.58 (−3.44, 0.28)
−1.99 (−3.84, −0.15)
group
healthy controls
AS cases
psychiatric controls
