# Supplementary material for: Empathy, psychopathology and suicidal behavior: a case–control study
Source: BMC Psychiatry. 2025 Aug 26;25:811. doi: 10.1186/s12888-025-07230-3 (PMC12379465; doi:10.1186/s12888-025-07230-3)
Supplement: Supplementary file 7 — Additional file 7. Predicted values of Perspective Taking (dependent variable) by SCL-90 psychological distress dimensions of Interpersonal Sensitivity, Depression, Anxiety and Hostility (main predictors) in AS cases, psychiatric controls and healthy controls. Results are from multivariate linear regression models adjusted for age, sex, civil status and professional level; each SCL-90 dimension was entered in separate regression models as main predictors. Numbers are unstandardized regression coefficients with 95% confidence intervals. [file 12888_2025_7230_MOESM7_ESM.pdf]

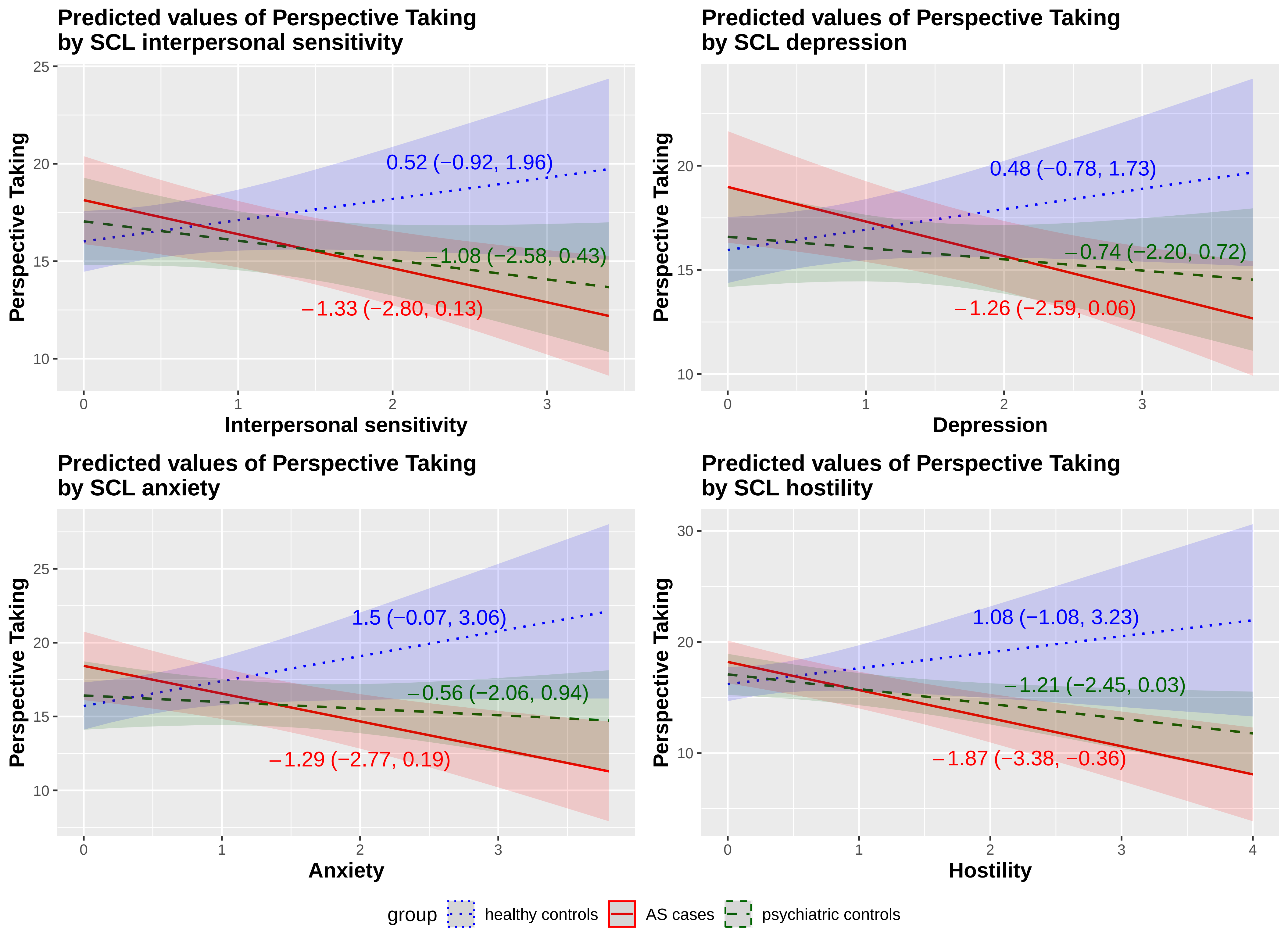
Predicted values of Perspective Taking by SCL interpersonal sensitivity
Perspective Taking
Interpersonal sensitivity
0.52 (−0.92, 1.96)
−1.08 (−2.58, 0.43)
−1.33 (−2.80, 0.13)
Predicted values of Perspective Taking by SCL depression
Perspective Taking
Depression
0.48 (−0.78, 1.73)
−0.74 (−2.20, 0.72)
−1.26 (−2.59, 0.06)
Predicted values of Perspective Taking by SCL anxiety
Perspective Taking
Anxiety
1.5 (−0.07, 3.06)
−0.56 (−2.06, 0.94)
−1.29 (−2.77, 0.19)
Predicted values of Perspective Taking by SCL hostility
Perspective Taking
Hostility
1.08 (−1.08, 3.23)
−1.21 (−2.45, 0.03)
−1.87 (−3.38, −0.36)
group
healthy controls
AS cases
psychiatric controls
