# Supplementary material for: Empathy, psychopathology and suicidal behavior: a case–control study
Source: BMC Psychiatry. 2025 Aug 26;25:811. doi: 10.1186/s12888-025-07230-3 (PMC12379465; doi:10.1186/s12888-025-07230-3)
Supplement: Supplementary file 8 — Additional file 8. Predicted values of Personal Distress (dependent variable) by SCL-90 psychological distress dimensions of Interpersonal Sensitivity, Depression, Anxiety and Hostility (main predictors) in AS cases, psychiatric controls and healthy controls. Results are from multivariate linear regression models adjusted for age, sex, civil status and professional level; each SCL-90 dimension was entered in separate regression models as main predictors. Numbers are unstandardized regression coefficients with 95% confidence intervals. [file 12888_2025_7230_MOESM8_ESM.pdf]

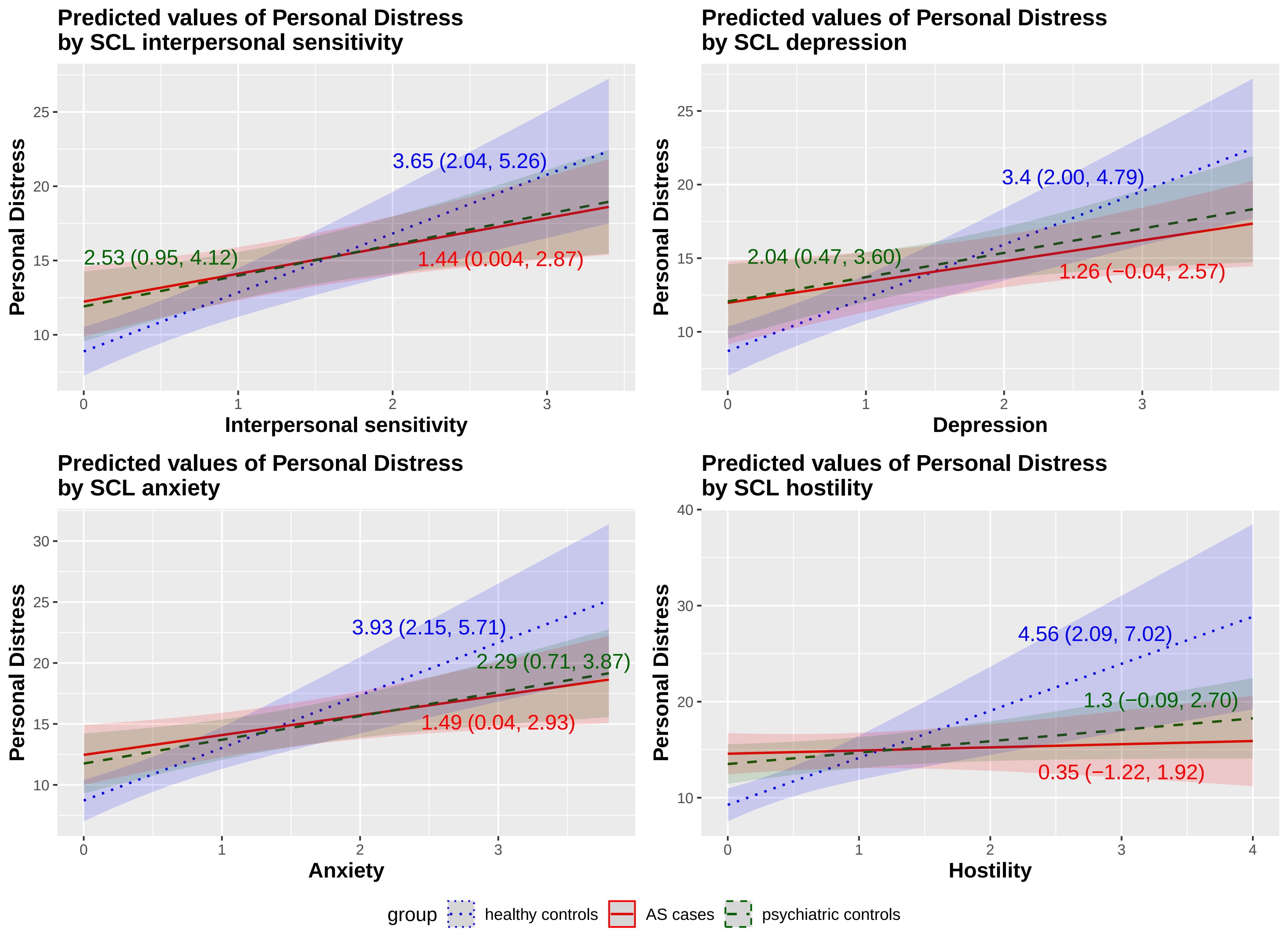
Predicted values of Personal Distress by SCL interpersonal sensitivity
3.65 (2.04, 5.26)
2.53 (0.95, 4.12)
1.44 (0.004, 2.87)
Personal Distress
Interpersonal sensitivity
Predicted values of Personal Distress by SCL depression
3.4 (2.00, 4.79)
2.04 (0.47, 3.60)
1.26 (−0.04, 2.57)
Depression
Predicted values of Personal Distress by SCL anxiety
3.93 (2.15, 5.71)
2.29 (0.71, 3.87)
1.49 (0.04, 2.93)
Anxiety
Predicted values of Personal Distress by SCL hostility
4.56 (2.09, 7.02)
1.3 (−0.09, 2.70)
0.35 (−1.22, 1.92)
Hostility
group
healthy controls
AS cases
psychiatric controls
