# Supplementary material for: Empathy, psychopathology and suicidal behavior: a case–control study
Source: BMC Psychiatry. 2025 Aug 26;25:811. doi: 10.1186/s12888-025-07230-3 (PMC12379465; doi:10.1186/s12888-025-07230-3)
Supplement: Supplementary file 9 — Additional file 9. Predicted values of Personal Distress (dependent variable) by SCL-90 psychological distress dimensions of Phobic Anxiety and Psychoticism (main predictors) in AS cases, psychiatric controls and healthy controls. Results are from multivariate linear regression models adjusted for age, sex, civil status and professional level; each SCL-90 dimension was entered in separate regression models as main predictors. Numbers are unstandardized regression coefficients with 95% confidence intervals. [file 12888_2025_7230_MOESM9_ESM.pdf]

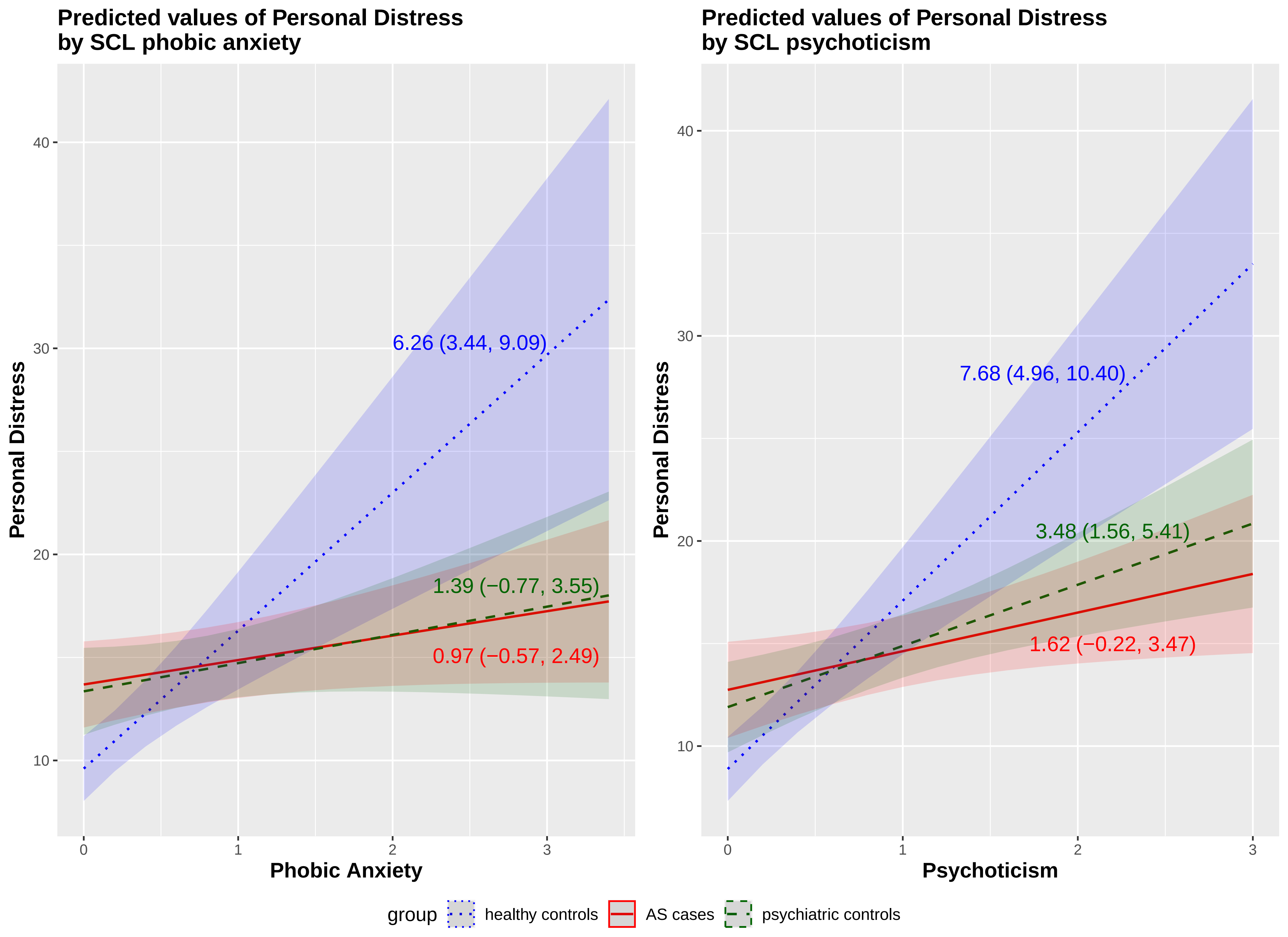

Predicted values of Personal Distress
by SCL phobic anxiety
Personal Distress
40
30
20
10
0
1
2
3
Phobic Anxiety
6.26 (3.44, 9.09)
1.39 (−0.77, 3.55)
0.97 (−0.57, 2.49)
Predicted values of Personal Distress
by SCL psychoticism
Personal Distress
40
30
20
10
0
1
2
3
Psychoticism
7.68 (4.96, 10.40)
3.48 (1.56, 5.41)
1.62 (−0.22, 3.47)
group
healthy controls
AS cases
psychiatric controls
